# Vaginal bacteria and cervical cancer: a bibliometric analysis of trends and themes

**DOI:** 10.3389/fmicb.2025.1615944

**Published:** 2025-07-30

**Authors:** Xiaoxia Liu, Yinghui Zhao, Xianhua Meng, Zheng Gao, Xiaohong Wang, Fengyong Yang

**Affiliations:** ^1^Department of Obstetrics and Gynecology, Jinan City People’s Hospital, People’s Hospital Affiliated to Shandong First Medical University, Jinan, Shandong, China; ^2^Department of Emergency, People's Hospital Affiliated to Shandong First Medical University, Jinan, China

**Keywords:** bibliometric analysis, bacterial vaginosis, cervical cancer, VOSviewer, CiteSpace

## Abstract

**Background:**

Cervical cancer remains a significant global health challenge, with increasing evidence suggesting the crucial role of vaginal bacteria in its development and progression. This study aims to analyze the global research landscape and trends in vaginal bacteria and cervical cancer research through bibliometric analysis.

**Methods:**

Literature data were retrieved from the Web of Science Core Collection (WOSCC) database. Bibliometric analysis was performed using VOSviewer, CiteSpace, and R-bibliometrix to evaluate publication patterns, research collaboration networks, and emerging trends.

**Results:**

A total of 372 publications were identified, showing an annual growth rate of 8.41%. China and USA emerged as leading contributors, with the Imperial College London and University of Arizona being the most productive institutions. Herbst-Kralovetz MM and Laniewski P were identified as the most influential authors, while *BMC Infectious Diseases* and *Frontiers in Cellular and Infection Microbiology* were the primary publication venues. Keyword co-occurrence analysis identified “bacterial vaginosis,” “women,” and “inflammation” as the most frequent terms, while burst detection revealed emerging research trends in “*lactobacillus*,” “intraepithelial neoplasia,” and “16 s rRNA gene sequencing.”

**Conclusion:**

This bibliometric analysis provides comprehensive insights into the evolution and current status of vaginal bacteria research in cervical cancer, highlighting key research themes and collaborative patterns. These findings offer valuable guidance for future research directions and potential clinical applications in cervical cancer prevention and treatment strategies.

## Introduction

Cervical cancer remains a significant global health challenge, ranking as the fourth most common cancer in women worldwide (Sung et al., 2021). In 2020, an estimated 604,000 new cases were diagnosed and 342,000 women died from the disease (Cao et al., 2024). The burden is particularly severe in low- and middle-income countries, which account for approximately 90% of cervical cancer deaths (Arbyn et al., 2020). Despite advances in prevention and treatment, the disease continues to pose substantial healthcare and socioeconomic challenges, significantly impacting patients’ quality of life. Early detection and appropriate treatment significantly influence cervical cancer prognosis. The standard screening approach combines cervical cytology and HPV testing (Perkins et al., 2023). Treatment strategies vary by disease stage, ranging from local excision for precancerous lesions to multimodal therapy incorporating surgery, radiotherapy, and chemotherapy for advanced cases, with 5-year survival rates declining from 92% for localized disease to 17% for metastatic cases (Cohen et al., 2019).

Despite these advances, significant challenges persist in understanding disease progression mechanisms. While HPV infection is necessary for cervical cancer development, only few infections progress to invasive cancer, suggesting crucial roles for additional cofactors (Schiffman and Wentzensen, 2013). Current research limitations include incomplete understanding of host–microbe interactions in disease progression (Leon-Gomez and Romero, 2025) and insufficient predictive biomarkers for identifying high-risk precancerous lesions (Holcakova et al., 2021). These knowledge gaps hinder the development of more effective, personalized treatment approaches and prevention strategies.

While persistent human papillomavirus (HPV) infection is recognized as the primary etiologic factor, recent evidence suggests that the vaginal bacteria plays a crucial role in disease development and progression (Mitra et al., 2016). Studies have demonstrated that vaginal microbial dysbiosis may influence HPV persistence and cervical carcinogenesis through various mechanisms. For instance, specific bacterial communities can alter local immune responses and epithelial barrier function, potentially creating conditions favorable for viral persistence and oncogenic transformation (Łaniewski et al., 2019). However, the complex interactions between vaginal microbiota, HPV infection, and cervical cancer development remain incompletely understood.

The vaginal bacteria has emerged as a potential biomarker for disease risk and progression, with studies demonstrating that specific microbial signatures may predict HPV persistence and cervical lesion development (Di Paola et al., 2017). Furthermore, microbiome-based therapeutic approaches, such as probiotics and microbiota modulation, are being investigated as novel treatment strategies (Norenhag et al., 2020). These advances suggest potential applications in disease screening, risk stratification, and personalized treatment approaches. However, the increasing volume and complexity of research make it challenging to identify key trends and emerging directions in this field.

Bibliometric analysis serves as a valuable tool for evaluating research impact and trends, offering quantitative insights into scientific literature development (Chen, 2017). Previous bibliometric studies have analyzed cervical cancer research from different perspectives, including global research trends in HPV and cervical cancer screening and early detection strategies (Khumalo et al., 2022) and treatment modalities (Duan et al., 2024). However, no comprehensive bibliometric analysis has yet examined the intersection of vaginal bacteria research and cervical cancer.

This study aims to provide the first systematic bibliometric analysis of research linking vaginal bacteria to cervical cancer, examining publication trends, research hotspots, and emerging directions. Understanding these patterns will help researchers identify promising research directions and potential collaborative opportunities, ultimately advancing our understanding of how vaginal microbiota influence cervical cancer development and progression.

## Materials and methods

### Data source and collection

The literature data analyzed in this study was sourced from the Web of Science Core Collection (WOSCC) database. WOSCC is a comprehensive citation database developed by Clarivate Analytics, which has been indexing the world’s most impactful research outputs since 1900, covering high-quality academic journals in natural sciences, social sciences, arts and humanities (Zhang et al., 2020). The search was conducted on November 6, 2024, with the following search strategy: (TS = ((genital OR vagin) AND (“microbiota” OR “microbiome” OR “vaginosis” OR “vaginoses” OR “vaginitis” OR “vaginitides” OR “lactobacill*” OR “monilia*” OR “*candida*” OR “*candidiasis*” OR “*candidosis*” OR “*gardnerella*”))) AND TS = (“cervical carcinoma” OR “cervical cancer” OR “cervical neoplasm” OR “cervical tumor” OR “cervical malignant”). The “Full Record and Cited References” format was chosen, and the selected literature was downloaded and saved as plain text files for subsequent analysis.

### Data analysis and visualization

Three analytical tools were employed for bibliometric analysis: VOSviewer (Version 1.6.20), R-bibliometrix (Version 4.3.3), and CiteSpace (Version 6.3. R1). The following data were extracted for analysis: publication counts, citations, titles, author information, institutions, countries/regions, keywords, and journal metrics.

VOSviewer was utilized to analyze collaboration networks and generate visualization maps of keyword co-occurrence (Arruda et al., 2022). In these visualizations, nodes represent individual elements (authors, institutions, countries, or keywords), with node size indicating frequency of occurrence. The connecting lines between nodes represent collaboration relationships or co-occurrence frequencies, with line thickness indicating the strength of these relationships. R-bibliometrix package was used to perform statistical analysis of scientific literature indices, including annual publication trends, country contributions, and journal impact metrics (Aria and Cuccurullo, 2017). CiteSpace was employed to identify research frontiers and emerging trends through burst detection analysis of keywords (Şahin and Yılmaz, 2022).

Several bibliometric indices were used to evaluate the academic impact of researchers and journals. The h-index, a measure of both the productivity and citation impact of a researcher, was calculated (Abbas, 2012). The g-index, which gives more weight to highly cited articles, was also computed (Egghe, 2006). Additionally, the m-index, calculated as the h-index divided by the number of years since the researcher’s first publication, was determined (Bažant and Nguyen, 2023). The Journal Citation Reports (JCR) 2023 was consulted to obtain journal impact factors (IF) and quartile rankings, where journals are classified into quartiles (Q1-Q4) based on their IF ranking within their respective subject categories (Kosyakov and Pislyakov, 2024; Rocha MOdC, 2013).

## Results

### Temporal development and publication characteristics

**Figure 1:**
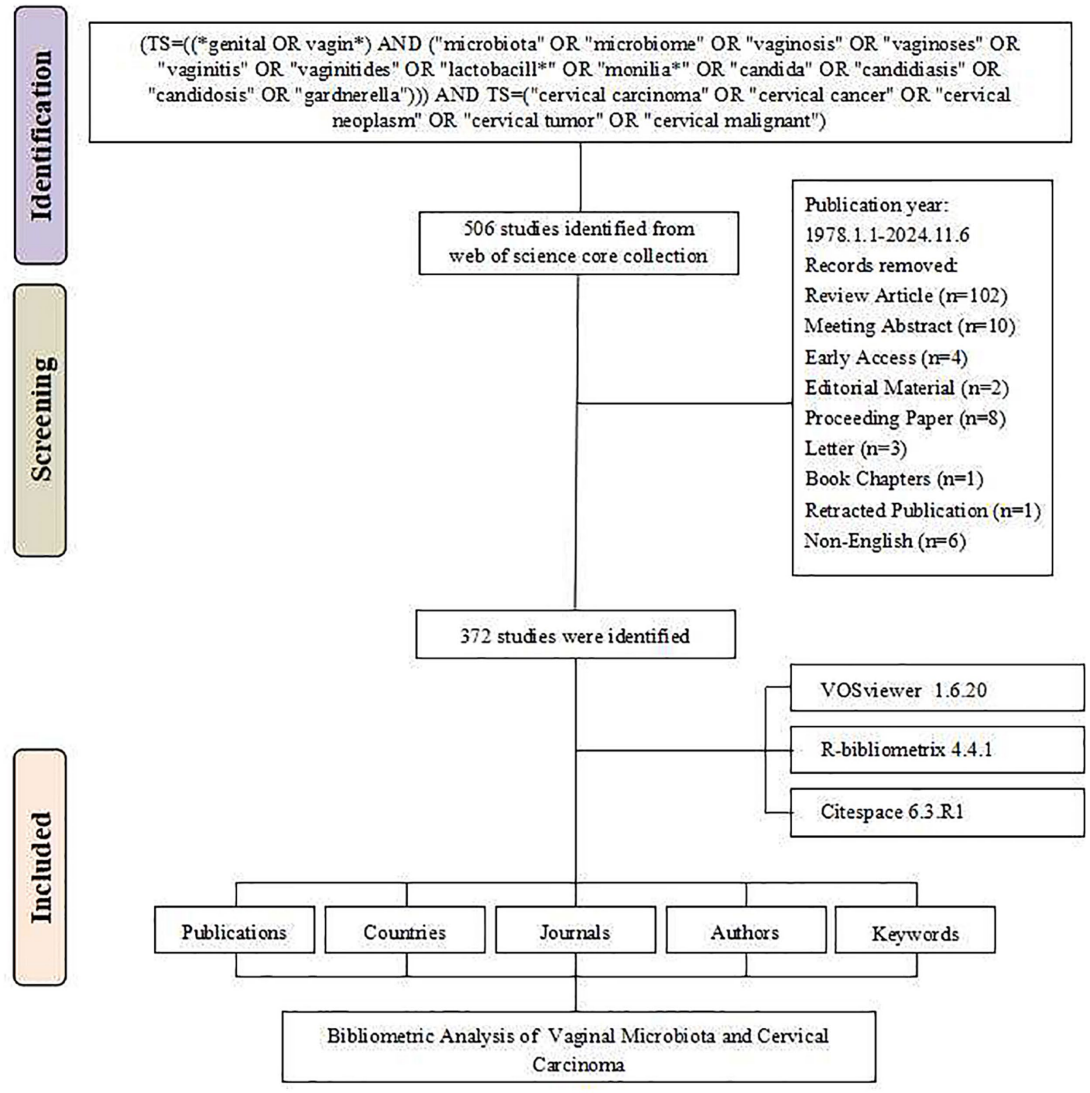
Literature screening flowchart for vaginal bacteria and cervical cancer research publications.

**Figure 2:**
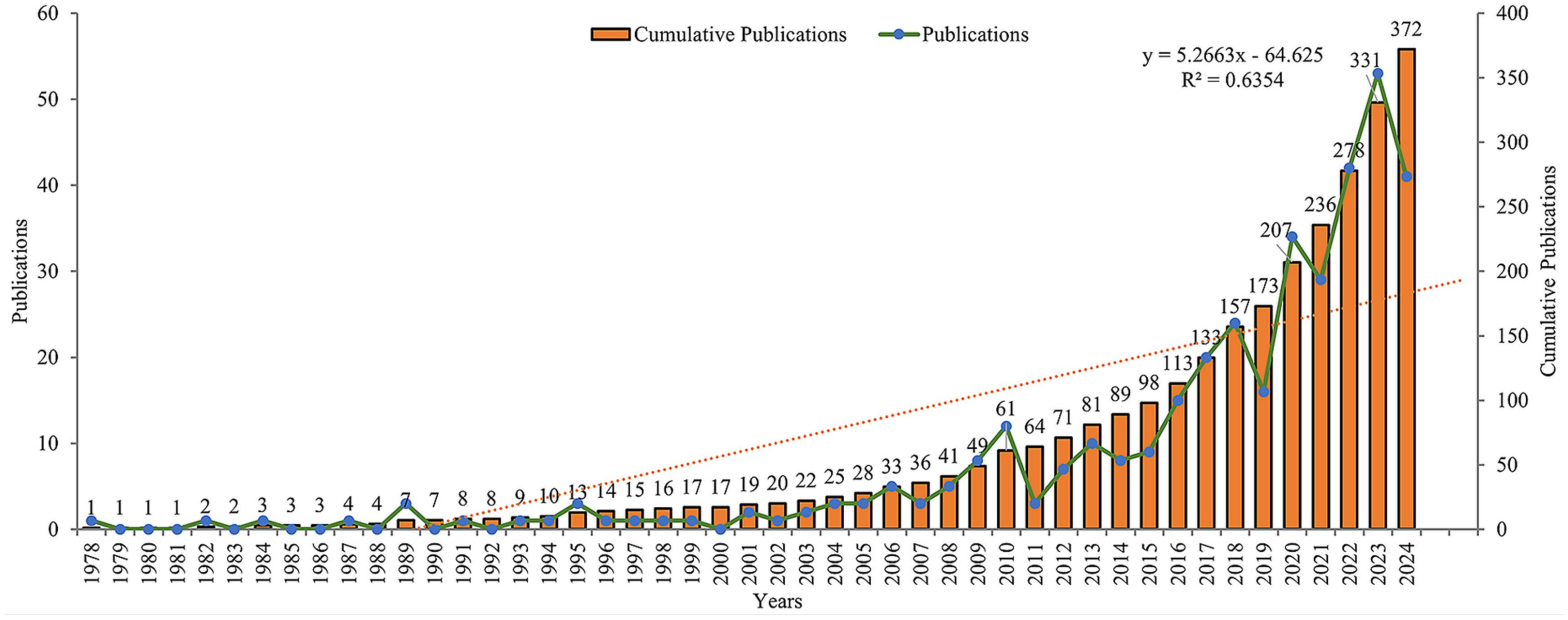
Annual publication trends of vaginal bacteria and cervical cancer research from 1978 to 2024.

According to our analysis of publication data from 1978 to 2024, this field has shown a continuous growth trend. The initial literature search identified 506 studies from the Web of Science Core Collection database. After systematic screening, 134 records were excluded based on the following criteria: review articles (*n* = 102), meeting abstracts (*n* = 10), early access publications (*n* = 4), editorial materials (*n* = 2), proceeding papers (*n* = 8), letters (*n* = 3), book chapters (*n* = 1), retracted publication (*n* = 1), and non-English publications (*n* = 6). The final analysis included 372 studies (Figure 1). The annual growth rate was 8.41%. During the initial stage spanning 1978–2000, research activity was relatively low, with annual publications generally remaining under 2 papers per year. The steady growth stage from 2001 to 2015 saw annual publications gradually increase, reaching around 10–15 papers per year. Finally, the rapid development stage from 2016 to 2024 witnessed a sharp increase in publications, with annual output exceeding 30 papers and reaching a peak of 53 papers in 2022. The cumulative number of publications reached 372 by 2024 (Figure 2).

The analysis identified 203 publication sources, with an international co-authorship rate of 23.12% and an average of 7.39 co-authors per document. Among the 2,436 contributing authors, only 8 papers were single-authored. The publications generated 10,393 references and averaged 21.46 citations per document, with a mean document age of 6.8 years. Author keyword analysis revealed 784 unique terms.

### Global research distribution and collaboration networks

**Figure 3:**
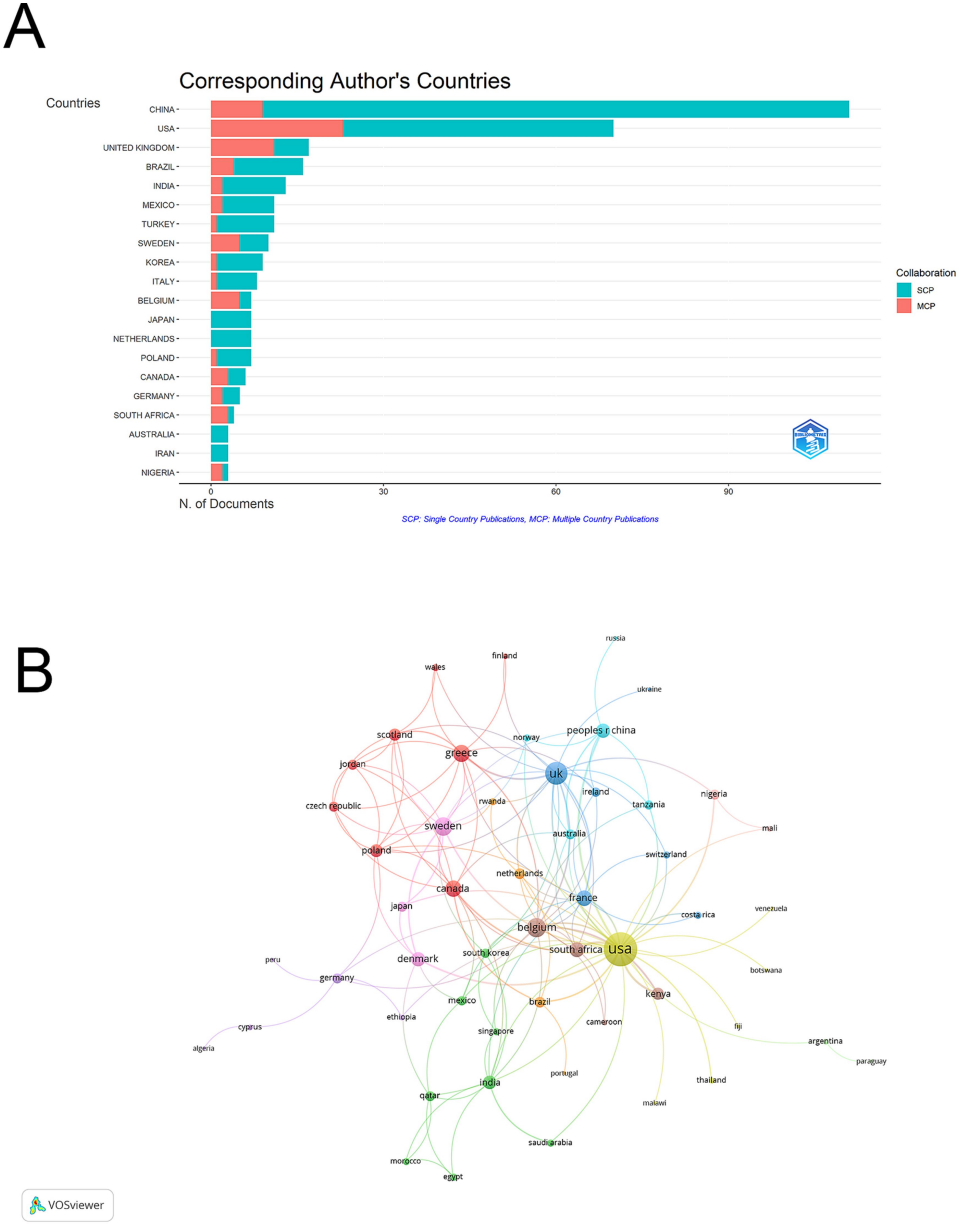
Geographic distribution and international collaboration network among vaginal bacteria and cervical cancer publications. **(A)** Global distribution map of publications by country/region. **(B)** International collaboration network visualization, where node size indicates publication volume and line thickness represents collaboration strength.

Based on bibliometric analysis of global research output, a total of 20 productive countries or regions contributed to vaginal bacteria and cervical cancer research (Supplementary Table 1; Figure 3A). China emerged as the leading contributor with 111 articles (29.8% of total publications), followed by USA with 70 articles (18.8%). UK ranked third with 17 articles (4.6%). Analysis of international collaboration patterns revealed different cooperation intensities among countries. South Africa demonstrated the highest international collaboration rate with an MCP ratio of 0.75, followed by Belgium (0.714) and Nigeria (0.667). In terms of citation impact, USA accumulated the highest total citations (2328), ranking first in citation performance. Belgium achieved the highest average citations per paper (95.3), while China, despite having the most publications, averaged 9.7 citations per paper.

The visualization network map illustrated that USA acted as a central hub for international collaboration with the highest total link strength of 66, forming strong cooperative links with European countries and China. The UK demonstrated the second strongest international collaboration network with a total link strength of 30, followed by Belgium (21) and Sweden (20). Among Asian countries, China showed moderate international connectivity with a total link strength of 12, while Japan had relatively limited international collaboration (total link strength = 6). Major European countries like France (15), Greece (17), and Denmark (12) maintained stable collaborative networks. This pattern suggests that while European countries formed interconnected research clusters, Asian countries showed relatively independent research patterns with lower international collaboration intensity (Figure 3B).

### Institutional research capacity and partnership patterns

**Figure 4:**
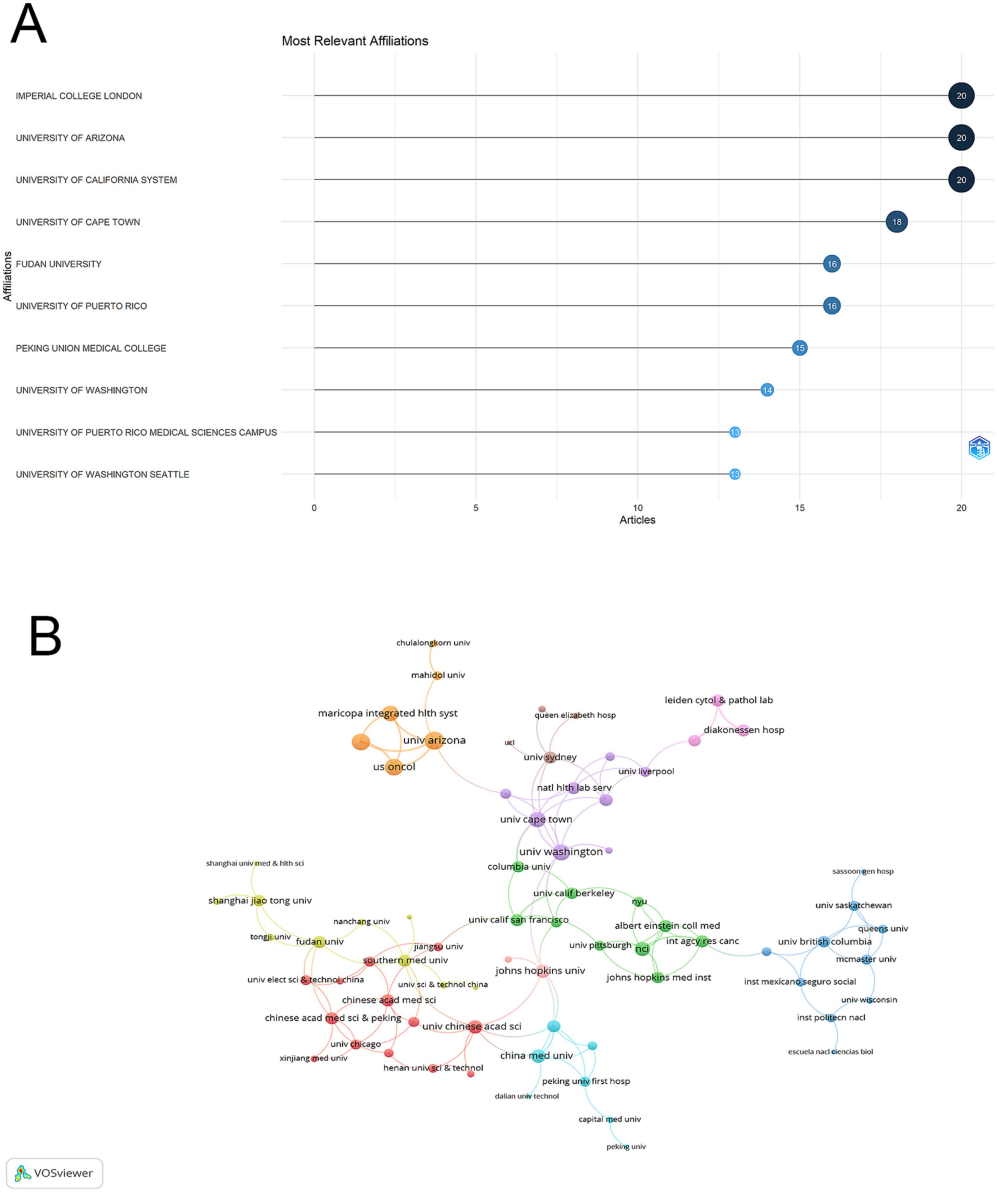
Institutional analysis and collaboration patterns in vaginal bacteria and cervical cancer. **(A)** Distribution of publications among top 10 contributing institutions. **(B)** Inter-institutional collaboration network visualization, with node size representing publication count and connecting lines indicating collaborative relationships.

The Imperial College London, University of Arizona, and University of California System emerged as the leading institutions, each contributing 20 publications to the field. The University of Cape Town followed with 18 publications, while Fudan University and University of Puerto Rico each produced 16 publications. Among Chinese institutions, Peking Union Medical College made notable contributions with 15 publications (Figure 4A).

The visualization network revealed distinct color-coded institutional clusters. The orange cluster was centered around University of Arizona, forming strong linkages with Maricopa Integrated Health System and US Oncology. The green cluster was dominated by University of California institutions and their affiliated campuses. The red cluster predominantly consisted of Chinese institutions, including Chinese Medical Sciences institutions and Fudan University. The purple cluster included institutions like University of Cape Town and University of Washington, while the light blue cluster was centered around China Medical University. In terms of collaboration strength, the University of Arizona demonstrated the highest connectivity (total link strength = 13), followed by Dignity Health St. Joseph’s Hospital & Medical Center and US Oncology (both with total link strength = 11). Imperial College London, Lund University, and University of Washington each showed strong collaborative ties (total link strength = 10). Among Chinese institutions, China Medical University (total link strength = 7) and various Chinese Academy institutions (total link strength = 6) displayed moderate connectivity. While regional collaborations were strong within clusters, cross-regional partnerships were also evident, particularly between North American and European institutions, though Chinese institutions showed relatively limited international connectivity despite their strong internal collaboration patterns (Figure 4B).

### Research leadership and collaborative research groups

Herbst-Kralovetz MM and Laniewski P emerged as leading contributors, each with an h-index of 7 and 436 total citations. Zhang Y demonstrated the highest productivity with 8 total publications (TP) and ranked first in TP rank, though ranking tenth in total citations with 115. Kyrgiou M, despite having fewer publications (TP = 5), achieved the highest citation impact with 903 total citations (Supplementary Table 2).

**Figure 5:**
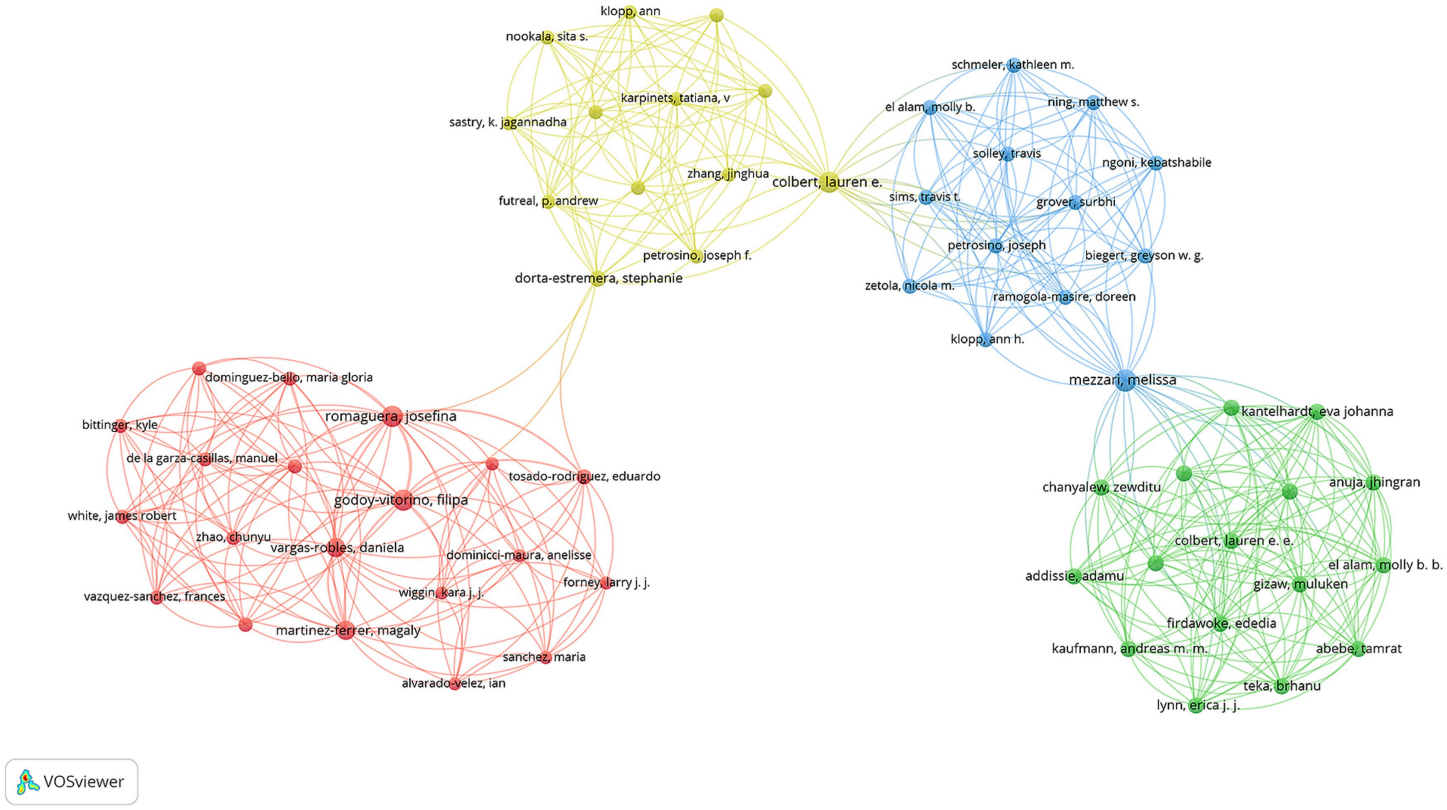
Author collaboration network visualization in vaginal bacteria and cervical cancer.

The visualization network map demonstrates four distinct color-coded collaboration clusters. Among these, three researchers—Apresa-Garcia Teresa, Lopez-Romero Ricardo, and Salcedo Mauricio—showed the highest collaboration intensity (total link strength = 35 each). The next tier of collaborative strength included Mezzari Melissa and Xue Fengxia (total link strength = 29 each). A group of researchers including Bekker Linda-Gail, Godoy-Vitorino Filipa, Kyrgiou M, Paraskevaidis E, Passmore Jo-Ann S, and Romaguera Josefina all demonstrated strong connectivity (total link strength = 26 each) (Figure 5).

### Publication venues and knowledge dissemination patterns

**Figure 6:**
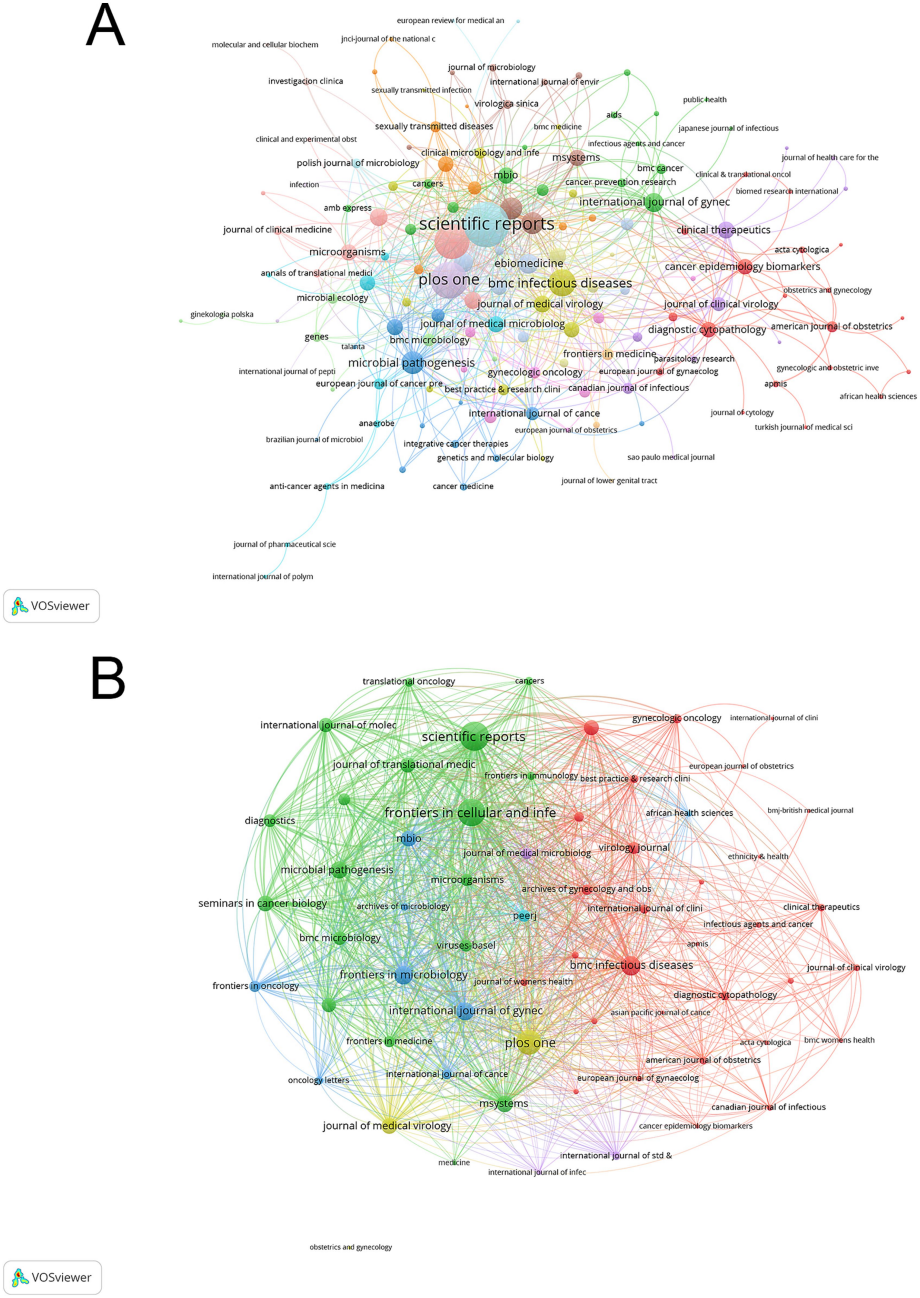
Journal analysis network in vaginal bacteria and cervical cancer. **(A)** Distribution and relationship of publishing journals based on citation patterns. **(B)** Journal bibliographic coupling network showing research theme clusters.

The journal co-occurrence network revealed four distinct clusters differentiated by colors. *BMC Infectious Diseases*, *Frontiers in Cellular and Infection Microbiology*, and *PLOS ONE* led with the highest h-indices of 8. *The American Journal of Obstetrics and Gynecology* showed the highest impact factor of 8.7, followed by *Journal of Medical Virology* (6.8) and *Frontiers in Cellular and Infection Microbiology* (4.6). In terms of publication volume, *Frontiers in Cellular and Infection Microbiology* and *PLOS ONE* shared the highest output with 15 papers each. *PLOS ONE* garnered the highest total citations (517), followed by *Scientific Reports* (368) (Supplementary Table 3). The earliest contributing journal was *the Journal of Medical Microbiology*, which began publishing relevant research in 1982. The visualization network in Figure 6 shows four main journal clusters, with *Scientific Reports*, *PLOS ONE*, and *BMC Infectious Diseases* forming central nodes within their respective clusters. The citation links between journals demonstrate that biomedical and clinical medicine journals served as primary knowledge sources in this field.

### Most cited publications

Analysis of citation impact identified several landmark papers that have shaped the field (Supplementary Table 4). The most cited article (511 citations, TC per year 30.06) was published in *BMJ* in 2008, titled “Perinatal mortality and other severe adverse pregnancy outcomes associated with treatment of cervical intraepithelial neoplasia: meta-analysis” (Arbyn et al., 2008). The second most cited paper (304 citations, TC per year 30.40), “Cervical intraepithelial neoplasia disease progression is associated with increased vaginal microbiome diversity,” was published in *Scientific Reports* in 2015 (Mitra et al., 2015). The third highest cited work (280 citations, TC per year 40.00), “The natural history of human papillomavirus infection,” appeared in *Best Practice & Research Clinical Obstetrics & Gynaecology* in 2018 (de Sanjose et al., 2018).

### Thematic evolution and research focus areas

**Figure 7:**
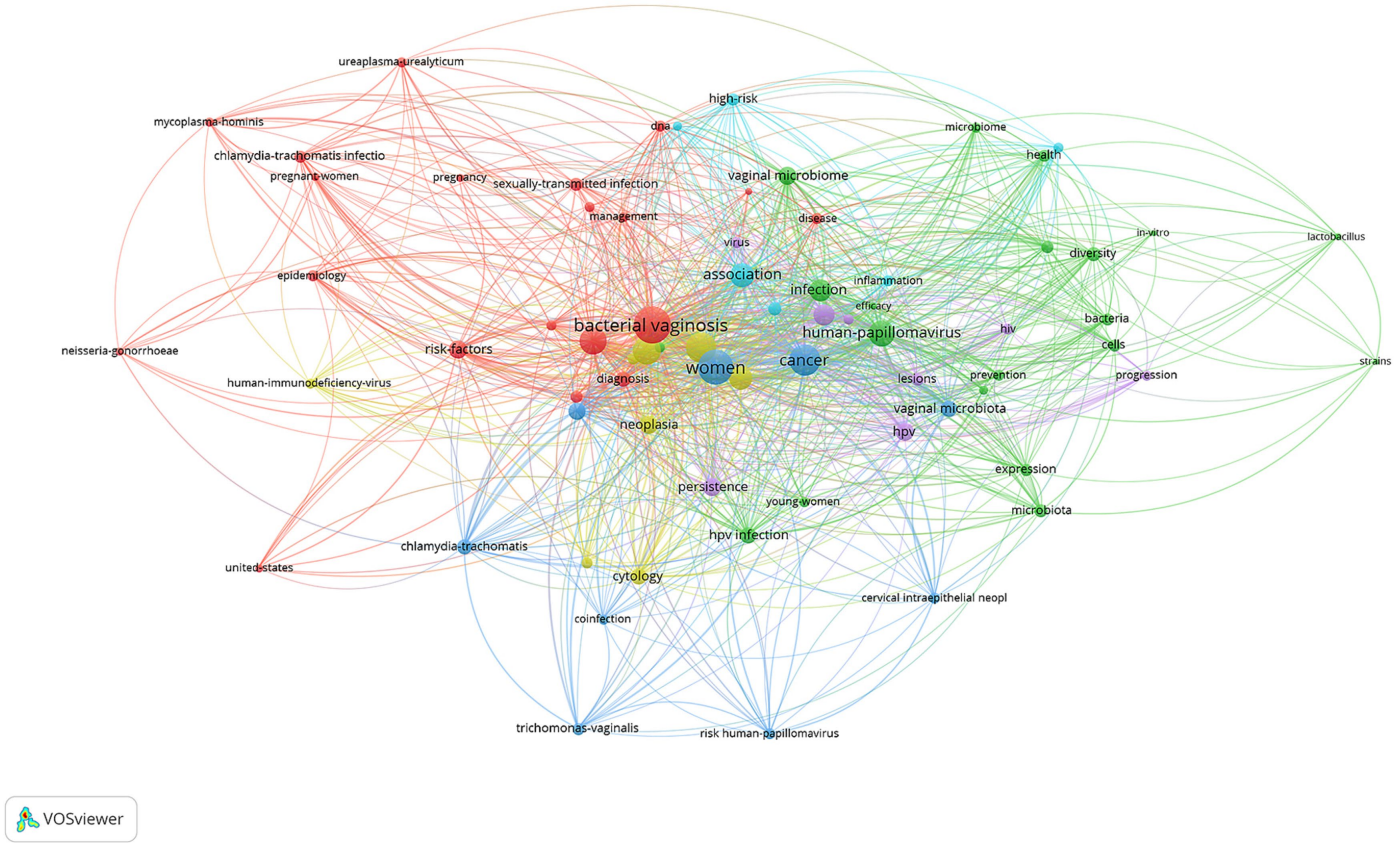
Keywords analysis in vaginal bacteria and cervical cancer.

The keyword co-occurrence network reveals distinct research themes in vaginal bacteria and cervical cancer studies. The analysis identified “bacterial vaginosis” (108 occurrences, link strength 439), “women” (94 occurrences, link strength 398), and “cancer” (73 occurrences, link strength 306) as the most prominent terms. The visualization software classified keywords into six thematic clusters (Figure 7A): Cluster 1 (Clinical Epidemiology and Risk Assessment, red, 20 items) focused on “bacterial vaginosis,” epidemiology and clinical aspects, with key terms including “risk factors,” “pregnant women” and “ureaplasma urealyticum”; Cluster 2 (Microbial Community Dynamics, green, 20 items) concentrated on “microbiome” characteristics, featuring “diversity,” “bacteria” and “*lactobacillus*”; Cluster 3 (Cervical Disease Progression, blue, 9 items) covered “cervical cancer” and related conditions, including “neoplasia,” “persistence” and “cytology”; Cluster 4 (Diagnostic and Detection Methods, yellow, 8 items) emphasized diagnostic aspects, with terms like “diagnosis,” “coinfection” and “trichomonas vaginalis”; Cluster 5 (Viral-Host Interactions, purple, 8 items) focused on viral factors, including “human papillomavirus,” “hiv” and “lesions”; and Cluster 6 (Inflammatory Pathways, light blue, 6 items) addressed inflammatory conditions, featuring terms such as “inflammation,” “high risk” and “disease.” These clusters demonstrate the multifaceted nature of research in this field, encompassing microbial, clinical, and pathological aspects of vaginal health and cervical cancer.

**Figure 8:**
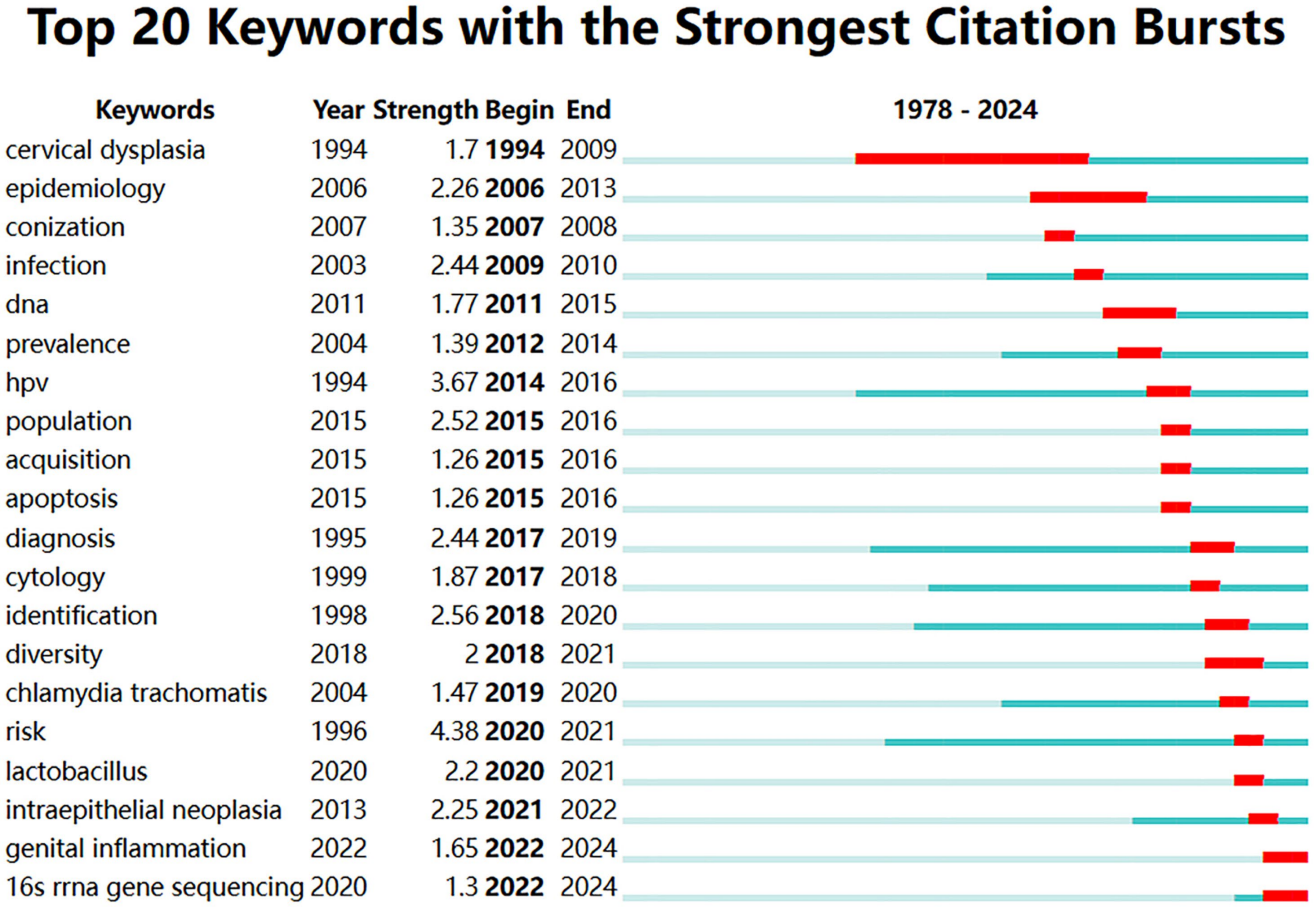
Timeline view of the top 20 keywords with the strongest citation bursts from 1978 to 2024.

The citation burst analysis (Figure 7B) revealed the evolution of research hotspots from 1978 to 2024. Early research emphasis was on “cervical dysplasia” (1994–2009, strength 1.7) and “epidemiology” (2006–2013, strength 2.26). Mid-period bursts included “infection” (2009–2010, strength 2.44) and “DNA” (2011–2015, strength 1.77). During 2014–2016, there was significant interest in “HPV” (strength 3.67) and related terms. Recent burst keywords (2020–2024) included “*lactobacillus*” (strength 2.2), “intraepithelial neoplasia” (strength 2.25), “genital inflammation” (strength 1.65), and “16 s rRNA gene sequencing” (strength 1.3) (Figure 8).

## Discussion

This bibliometric analysis reveals significant growth in vaginal bacteria and cervical cancer research from 1978 to 2024, with 372 publications and an 8.41% annual growth rate. The field shows increasing international collaboration, evidenced by a 23.12% international co-authorship rate and an average of 7.39 co-authors per document. The three most cited papers reflect the field’s key research priorities: clinical outcome assessment of cervical treatments (511 citations), microbiome diversity in disease progression (304 citations), and HPV infection natural history (280 citations), demonstrating the evolution from clinical observations to mechanistic understanding of microbiome-disease relationships.

Global research contributions show distinct regional patterns. High and upper-middle-income countries, such as China and USA, lead publication output, reflecting their substantial investment in women’s health research infrastructure (Fung et al., 2015), although most of the cervical cancer cases are in the low- and middle-income countries (Arbyn et al., 2020). Consequenly, progress in terms of a reduction in cervical cancer incidence and mortality, has thus far been observed predominantly in high and upper-middle-income countries, where high-quality screening, timely treatment, and follow-up care services are routinely available (He and Li, 2021). Besides, incidence of cervical cancer was three times higher in countries with low Human Development Index (HDI) than countries with very high HDI, whereas mortality rates were six times higher in low HDI countries versus very high HDI countries (Singh et al., 2023).

The higher citation impact of U. S. publications compared to Chinese publications suggests differences in research focus and international visibility. The institutional analysis reveals concentrated expertise in specific regions. The University of California System’s prominent role aligns with its historical strengths in microbiome research and gynecologic oncology (Knight et al., 2018). Chinese institutions, led by Peking Union Medical College, demonstrate substantial contributions but relatively isolated collaboration patterns, indicating potential opportunities for enhanced international partnerships. Key researchers have shaped distinct research directions. Herbst-Kralovetz and Laniewski’s high citation rates reflect their pioneering work in vaginal bacteria characterization (Hummelen et al., 2011), while Kyrgiou’s impact stems from systematic investigations of cervical cancer risk factors (Kyrgiou et al., 2017). The prominence of specialized journals reflects the field’s interdisciplinary nature. *BMC Infectious Diseases* and *Frontiers in Cellular and Infection Microbiology* have emerged as leading platforms, suggesting growing interest in microbiome-related research in oncology. This aligns with broader trends in microbiome research applications in cancer biology. The high impact factor of the *Journal of Medical Virology* indicates the importance of viral factors in cervical cancer pathogenesis, particularly regarding HPV infection mechanisms (Furumoto and Irahara, 2002).

The keyword co-occurrence analysis reveals complex research landscapes across these six clusters, with research focused primarily on establishing clinical associations within Cluster 1 (Clinical Epidemiology). Clinical studies demonstrated that women with bacterial vaginosis showed a 2–3 fold increased risk of acquiring sexually transmitted infections and developing cervical intraepithelial neoplasia (22). This fundamental finding was further supported by Castle et al. (27), who specifically quantified a 2.5-fold increased risk of cervical intraepithelial neoplasia (CIN2+), while Watts et al. (28) identified specific bacterial taxa associated with dysplastic progression.

A significant advancement in Cluster 2 (Microbial Community Dynamics) came when Ravel et al. (2011) characterized five community state types (CSTs) of vaginal microbiota: four dominated by different *Lactobacillus* species (CST I, II, III, V) and one diverse type (CST IV) featuring anaerobic bacteria characteristic of bacterial vaginosis. CST IV, marked by high proportions of *Gardnerella*, *Prevotella*, and other anaerobes with depleted *Lactobacillus*, was associated with increased risk of both HPV infection and cervical dysplasia (Di Paola et al., 2017). This taxonomic and clinical foundation was later expanded by mechanistic investigations.

The strong co-occurrence between Clusters 1 and 6 is evidenced by “bacterial vaginosis” linking with both “hpv” and “inflammation” in our network analysis, reflecting emerging understanding of how vaginal dysbiosis may create a microenvironment conducive to viral persistence and neoplastic transformation. This progression from clinical associations to molecular mechanisms represents a key advancement in understanding the vaginal bacteria’s role in cervical cancer development.

The network connections between Clusters 2 and 5 (Viral-Host Interactions) reveals an interesting dichotomy in research approaches. While some studies focus on direct viral-bacterial interactions, others examine broader ecological disruptions. Łaniewski et al. (2019) found that HPV infection correlates with increased microbial diversity and decreased *Lactobacillus* abundance, specifically noting a reduction in *L. crispatus* and *L. jensenii*. Di Paola et al. (2017) demonstrated that *L. crispatus* depletion, rather than overall community diversity, strongly correlates with HPV persistence, as this species produces high levels of D-lactic acid maintaining vaginal pH below 4.5. These seemingly conflicting findings were reconciled by Champer et al. (2018), who showed that while *L. crispatus* provides direct antiviral effects through lactic acid production, overall community stability prevents the overgrowth of anaerobes that produce harmful metabolites.

The research themes in Cluster 6 (Inflammatory Pathways) reveals complex microbe-host interactions mediated by specific bacterial metabolites. Valenti et al. (2018) identified that BV-associated bacteria, particularly *Gardnerella vaginalis* and *Prevotella bivia*, produce short-chain fatty acids (primarily succinate and acetate) and polyamines (cadaverine, putrescine) that significantly alter the local immune environment. These metabolites trigger NOD-like receptor activation in cervical epithelial cells, leading to increased production of pro-inflammatory cytokines IL-1β, IL-6, and IL-8. Mitra et al. (2016) further demonstrated that these inflammatory patterns correlate with disease progression, showing that women with cervical intraepithelial neoplasia grade 2 + (CIN2+) had significantly elevated levels of IL-1β (2.3-fold) and TNF-*α* (1.8-fold) compared to controls. Additionally, they found that succinate-producing bacteria were enriched 3.5-fold in CIN2 + cases.

The bidirectional relationship between inflammation and microbial composition, bridging Clusters 2 and 6, was elucidated by Shannon et al. (2017), who showed that pre-existing inflammatory conditions, characterized by elevated pro-inflammatory cytokines (particularly IL-1β > 200 pg./mL), significantly reduced *Lactobacillus* abundance while promoting the growth of anaerobes. This creates a feedback loop where inflammation-induced changes in the microbial community lead to increased production of pro-inflammatory metabolites, further perpetuating the inflammatory state. Specifically, they observed that sustained inflammation resulted in a 4-fold increase in *Gardnerella vaginalis* and a 3-fold increase in *Atopobium vaginae* abundance, while reducing *L. crispatus* levels by 60%.

The strong co-occurrence of “bacterial vaginosis” with both “hpv” and “inflammation” in our network analysis reflects this emerging understanding of how vaginal dysbiosis may create a microenvironment conducive to viral persistence and neoplastic transformation. This progression from clinical associations to molecular mechanisms represents a key advancement in understanding the vaginal bacteria’s role in cervical cancer development.

In a further step, the temporal analysis of keyword bursts reveals the evolution of vaginal bacteria research in cervical cancer from initial pathological observations to mechanistic understandings. The temporal evolution of keyword bursts reveals the progression of research focus in this field.

The temporal analysis shows how research evolved across these clusters, particularly in Cluster 4 (Diagnostic and Detection Methods). The “epidemiology” burst marked the transition to population-level investigations of vaginal bacteria patterns in cervical disease. Stoler et al. (1992) conducted a meta-analysis of 12 studies involving 6,372 women, establishing a significant association between bacterial vaginosis and HPV infection (pooled OR 1.43, 95% CI 1.11–1.84). This period also saw the emergence of large-scale studies characterizing vaginal bacteria profiles across diverse populations, with Arbyn et al. (2011) identifying five distinct community state types (CSTs) and their associations with cervical health outcomes.

A significant advancement occurred during 2014–2016, when “HPV” showed an intense burst, coinciding with deeper investigations into virus-microbiome interactions. Franceschi et al. (2009) revealed that HPV-positive women showed a 2-fold increase in non-*Lactobacillus*-dominant communities compared to HPV-negative controls. Sankaranarayanan et al. (2009) further demonstrated that persistent HPV infection was associated with a 3.5-fold decrease in *L. crispatus* abundance and increased anaerobic diversity. Following these epidemiological observations, mechanistic studies revealed *L. crispatus* as a key protective species, operating through multiple pathways including bacteriocin production, epithelial barrier maintenance, and pH regulation. These molecular mechanisms collectively establish *Lactobacillus* species as promising therapeutic targets in cervical disease prevention (Doorbar et al., 2015).

The emergence of “16 s rRNA gene sequencing” (2022–2024) alongside “genital inflammation” (2022–2024) has enabled unprecedented resolution in microbiome-immune interaction studies. Multi-omic analyses combining 16S sequencing with metabolomics have identified specific microbial signatures associated with cervical health and disease states. Mitra et al. (2016) used this approach to demonstrate that bacterial vaginosis-associated anaerobes, precisely identified through 16S sequencing, produce specific metabolites that may promote oncogenesis through DNA damage pathways. These metabolites were shown to decrease expression of tumor suppressor proteins and increase inflammatory mediators in cervical epithelial cells (Wentzensen et al., 2016; Thomas et al., 2024).

The “intraepithelial neoplasia” (2021–2022) research trend highlights the crucial pre-cancerous stage in cervical cancer development. Cervical intraepithelial neoplasia (CIN) represents a spectrum of pre-malignant changes, with approximately 10–15% of high-grade lesions progressing to invasive cancer if left untreated (Schiffman et al., 2007). Studies have demonstrated that the progression from low-grade (CIN1) to high-grade lesions (CIN2/3) is associated with specific shifts in microbial communities. An analysis of 273 women by Mitra et al. (2015) found that increasing CIN severity correlates with decreasing *Lactobacillus* abundance and increasing microbial diversity. Similar findings were reported by Oh et al. (2015), who observed that women with CIN2 + showed significant reductions in *L. crispatus* compared to those with normal cytology. Citation analysis reveals a clear progression from basic science discoveries to clinical validation studies, with increasing focus on identifying microbiome signatures that could predict CIN progression risk. The impact of this research is reflected in clinical practice, with recent cervical cancer screening guidelines incorporating microbiome-based assessments as complementary tools for CIN management (Gao et al., 2023; Ni et al., 2024).

The temporal evolution of keyword bursts in vaginal bacteria research reflects a remarkable progression from descriptive studies to sophisticated mechanistic understanding. The transition from early keywords like “cervical dysplasia” (1994–1999) through “epidemiology” (2006–2013) to recent bursts in “*Lactobacillus*” (2020–2021) and “16 s rRNA gene sequencing” (2022–2024) demonstrates the field’s maturation. This progression has revealed critical insights into microbiome-host interactions, particularly the protective mechanisms of *Lactobacillus* species and their metabolites in cervical health. The convergence of multiple research directions, evidenced by concurrent bursts in inflammation-related terms and “intraepithelial neoplasia” (2021–2022), has established a comprehensive framework linking microbial communities, host immunity, and disease progression. These advances have not only enhanced our fundamental understanding but also opened promising avenues for microbiome-based therapeutic interventions and diagnostic strategies in cervical cancer prevention and treatment.

### Strengths and limitations

This study offers significant strengths through its comprehensive bibliometric analysis spanning multiple decades, providing a longitudinal perspective on research evolution, and its integration of citation analysis with content review, enabling identification of both influential works and emerging research frontiers. However, several limitations should be noted: the reliance on citation counts as a metric of impact may underestimate the clinical significance of recently published works or practice-changing studies that have not yet accumulated substantial citations, and the exclusion of non-English publications and gray literature might have omitted relevant research contributions, particularly from regions with significant disease burden but limited English-language publications. Additionally, although our search strategy included broad terms to maximize bacterial, some species may have been missed. Similarly, cervical intraepithelial neoplasia, a kind of precancerous lesions of cervical cancer, was miss in our search strategy, which may introduce the bias into the results. Given the limited literature on non-HPV microbes in cervical cancer, this omission likely does not affect overall bibliometric trends.

## Conclusion

This bibliometric analysis of vaginal bacteria and cervical cancer research (1978–2024) identified 372 publications. Research evolved from descriptive studies to mechanistic investigations, revealing six major research clusters: clinical epidemiology, microbial community dynamics, disease progression, diagnostic methods, viral-host interactions, and inflammatory pathways. Key research trends progressed from basic bacterial vaginosis studies to sophisticated analyses of *Lactobacillus* protective mechanisms and inflammatory responses. These findings highlight promising directions for microbiome-based approaches in cervical cancer prevention, diagnosis, and treatment, offering valuable guidance for future research and clinical applications.

## Data Availability

The original contributions presented in the study are included in the article/Supplementary material, further inquiries can be directed to the corresponding authors.

## References

[ref1] Abbas A. M. (2012). Bounds and inequalities relating h-index, g-index, e-index and generalized impact factor: an improvement over existing models. PLoS One 7:e33699. doi: 10.1371/journal.pone.0033699, PMID: 22496760 PMC3319552

[ref2] Arbyn M. Castellsagué X. de Sanjosé S. Bruni L. Saraiya M. Bray F. . (2011). Worldwide burden of cervical cancer in 2008. Ann. Oncol. 22, 2675–2686. doi: 10.1093/annonc/mdr015, PMID: 21471563

[ref3] Arbyn M. Kyrgiou M. Simoens C. Raifu A. Koliopoulos G. Martin-Hirsch P. . (2008). Perinatal mortality and other severe adverse pregnancy outcomes associated with treatment of cervical intraepithelial neoplasia: meta-analysis. BMJ 337:a1284. doi: 10.1136/bmj.a1284, PMID: 18801868 PMC2544379

[ref4] Arbyn M. Weiderpass E. Bruni L. de Sanjosé S. Saraiya M. Ferlay J. . (2020). Estimates of incidence and mortality of cervical cancer in 2018: a worldwide analysis. Lancet Glob. Health 8, e191–e203. doi: 10.1016/S2214-109X(19)30482-6, PMID: 31812369 PMC7025157

[ref5] Aria M. Cuccurullo C. (2017). Bibliometrix: an R-tool for comprehensive science mapping analysis. J. Informetr. 11, 959–975. doi: 10.1016/j.joi.2017.08.007

[ref6] Arruda H. Silva E. R. Lessa M. Proença D. Jr. Bartholo R. (2022). VOSviewer and bibliometrix. J. Med. Libr. Assoc. 110, 392–395. doi: 10.5195/jmla.2022.1434, PMID: 36589296 PMC9782747

[ref7] Bažant Z. P. Nguyen H. T. (2023). Proposal of m-index for rating fracture and damage models by their ability to represent a set of distinctive experiments. J. Eng. Mech. 149:04023047. doi: 10.1061/JENMDT.EMENG-6887

[ref8] Cao W. Qin K. Li F. Chen W. (2024). Comparative study of cancer profiles between 2020 and 2022 using global cancer statistics (GLOBOCAN). J. Natl. Cancer Center 4, 128–134. doi: 10.1016/j.jncc.2024.05.001, PMID: 39282581 PMC11390618

[ref9] Champer M. Wong A. Champer J. Brito I. Messer P. Hou J. . (2018). The role of the vaginal microbiome in gynaecological cancer. BJOG Int. J. Obstet. Gynaecol. 125, 309–315. doi: 10.1111/1471-0528.14631, PMID: 28278350

[ref10] Chen C. (2017). Science mapping: a systematic review of the literature. J. Data Inf. Sci. 2, 1–40. doi: 10.1515/jdis-2017-0006

[ref11] Cohen P. A. Jhingran A. Oaknin A. Denny L. (2019). Cervical cancer. Lancet 393, 169–182. doi: 10.1016/S0140-6736(18)32470-X, PMID: 30638582

[ref12] de Sanjose S. Brotons M. Pavon M. A. (2018). The natural history of human papillomavirus infection. Best Pract. Res. Clin. Obstet. Gynaecol. 47, 2–13. doi: 10.1016/j.bpobgyn.2017.08.015, PMID: 28964706

[ref13] Di Paola M. Sani C. Clemente A. M. Iossa A. Perissi E. Castronovo G. . (2017). Characterization of cervico-vaginal microbiota in women developing persistent high-risk human papillomavirus infection. Sci. Rep. 7:10200. doi: 10.1038/s41598-017-09842-6, PMID: 28860468 PMC5579045

[ref14] Doorbar J. Egawa N. Griffin H. Kranjec C. Murakami I. (2015). Human papillomavirus molecular biology and disease association. Rev. Med. Virol. 25, 2–23. doi: 10.1002/rmv.1822, PMID: 25752814 PMC5024016

[ref15] Duan Y. Yang L. Wang W. Zhang P. Fu K. Li W. . (2024). A comprehensive bibliometric analysis (2000–2022) on the mapping of knowledge regarding immunotherapeutic treatments for advanced, recurrent, or metastatic cervical cancer. Front. Pharmacol. 15:1351363. doi: 10.3389/fphar.2024.1351363, PMID: 38799160 PMC11116801

[ref16] Egghe L. (2006). Theory and practise of the g-index. Scientometrics 69, 131–152. doi: 10.1007/s11192-006-0144-7

[ref17] Franceschi S. Plummer M. Clifford G. De Sanjose S. Bosch X. Herrero R. . (2009). Differences in the risk of cervical cancer and human papillomavirus infection by education level. Br. J. Cancer 101, 865–870. doi: 10.1038/sj.bjc.6605224 19654578 PMC2736843

[ref18] Fung I. C.-H. Hao Y. Cai J. Ying Y. Schaible B. J. Yu C. M. . (2015). Chinese social media reaction to information about 42 notifiable infectious diseases. PLoS One 10:e0126092. doi: 10.1371/journal.pone.0126092, PMID: 25946020 PMC4422708

[ref19] Furumoto H. Irahara M. (2002). Human papilloma virus (HPV) and cervical cancer. J. Med. Investig. 49, 124–133. doi: 10.3390/cells8060622 12323001

[ref20] Gao Q. Fan T. Luo S. Zheng J. Zhang L. Cao L. . (2023). *Lactobacillus gasseri* LGV03 isolated from the cervico-vagina of HPV-cleared women modulates epithelial innate immune responses and suppresses the growth of HPV-positive human cervical cancer cells. Transl. Oncol. 35:101714. doi: 10.1016/j.tranon.2023.101714, PMID: 37331103 PMC10366645

[ref21] He W. Q. Li C. (2021). Recent global burden of cervical cancer incidence and mortality, predictors, and temporal trends. Gynecol. Oncol. 163, 583–592. doi: 10.1016/j.ygyno.2021.10.075, PMID: 34688503

[ref22] Holcakova J. Bartosik M. Anton M. Minar L. Hausnerova J. Bednarikova M. . (2021). New trends in the detection of gynecological precancerous lesions and early-stage cancers. Cancers 13:6339. doi: 10.3390/cancers13246339, PMID: 34944963 PMC8699592

[ref23] Hummelen R. Macklaim J. M. Bisanz J. E. Hammond J.-A. McMillan A. Vongsa R. . (2011). Vaginal microbiome and epithelial gene array in post-menopausal women with moderate to severe dryness. PLoS One 6:e26602. doi: 10.1371/journal.pone.0026602, PMID: 22073175 PMC3206802

[ref24] Khumalo P. G. Carey M. Mackenzie L. Ampofo A. G. Sanson-Fisher R. (2022). Trends in cervical cancer screening research in sub-Saharan Africa: a bibliometric analysis of publications from 2001 to 2020. J. Cancer Policy 34:100356. doi: 10.1016/j.jcpo.2022.100356, PMID: 35995396

[ref25] Knight R. Vrbanac A. Taylor B. C. Aksenov A. Callewaert C. Debelius J. . (2018). Best practices for analysing microbiomes. Nat. Rev. Microbiol. 16, 410–422. doi: 10.1038/s41579-018-0029-9, PMID: 29795328

[ref26] Kosyakov D. Pislyakov V. (2024). “I'd like to publish in Q1, but there's no Q1 to be found”: study of journal quartile distributions across subject categories and topics. J. Informetr. 18:101494. doi: 10.1016/j.joi.2024.101494

[ref27] Kyrgiou M. Kalliala I. Markozannes G. Gunter M. J. Paraskevaidis E. Gabra H. . (2017). Adiposity and cancer at major anatomical sites: umbrella review of the literature. BMJ:j477. doi: 10.1136/bmj.j477, PMID: 28246088 PMC5421437

[ref28] Łaniewski P. Cui H. Roe D. J. Barnes D. Goulder A. Monk B. J. . (2019). Features of the cervicovaginal microenvironment drive cancer biomarker signatures in patients across cervical carcinogenesis. Sci. Rep. 9:7333. doi: 10.1038/s41598-019-43849-5, PMID: 31089160 PMC6517407

[ref29] Leon-Gomez P. Romero V. I. (2025). Human papillomavirus, vaginal microbiota and metagenomics: the interplay between development and progression of cervical cancer. Front. Microbiol. 15:1515258. doi: 10.3389/fmicb.2024.1515258, PMID: 39911706 PMC11794528

[ref30] Mitra A. Mac Intyre D. A. Marchesi J. R. Lee Y. S. Bennett P. R. Kyrgiou M. (2016). The vaginal microbiota, human papillomavirus infection and cervical intraepithelial neoplasia: what do we know and where are we going next? Microbiome 4, 1–15. doi: 10.1186/s40168-016-0203-0 27802830 PMC5088670

[ref31] Mitra A. MacIntyre D. A. Lee Y. Smith A. Marchesi J. R. Lehne B. . (2015). Cervical intraepithelial neoplasia disease progression is associated with increased vaginal microbiome diversity. Sci. Rep. 5:16865. doi: 10.1038/srep16865 26574055 PMC4648063

[ref32] Ni H. Huang C. Ran Z. Li S. Kuang C. Zhang Y. . (2024). Targeting HPV for the prevention, diagnosis, and treatment of cervical cancer. J. Mol. Cell Biol. 16:mjae046. doi: 10.1093/jmcb/mjae046, PMID: 39402008 PMC12080229

[ref33] Norenhag J. Du J. Olovsson M. Verstraelen H. Engstrand L. Brusselaers N. (2020). The vaginal microbiota, human papillomavirus and cervical dysplasia: a systematic review and network meta-analysis. BJOG Int. J. Obstet. Gynaecol. 127, 171–180. doi: 10.1111/1471-0528.15854, PMID: 31237400

[ref34] Oh H. Kim B.-S. Seo S.-S. Kong J.-S. Lee J.-K. Park S.-Y. . (2015). The association of uterine cervical microbiota with an increased risk for cervical intraepithelial neoplasia in Korea. Clin. Microbiol. Infect. 21:e671-674. e679:674. doi: 10.1016/j.cmi.2015.02.026 25752224

[ref35] Perkins R. B. Wentzensen N. Guido R. S. Schiffman M. (2023). Cervical cancer screening: a review. JAMA 330, 547–558. doi: 10.1001/jama.2023.13174, PMID: 37552298

[ref36] Ravel J. Gajer P. Abdo Z. Schneider G. M. Koenig S. S. McCulle S. L. . (2011). Vaginal microbiome of reproductive-age women. Proc. Natl. Acad. Sci. USA 108, 4680–4687. doi: 10.1073/pnas.1002611107, PMID: 20534435 PMC3063603

[ref37] Rocha MOdC (2013). Author recognition, impact factor, relevance, and the meaning of publishing. Brasil: Sci ELO Brasil, 125–127. 10.1590/0037-8682-0070-2013 23740061

[ref38] Şahin A. Yılmaz G. (2022). Local food research: a bibliometric review using CiteSpace II (1970–2020). Libr. Hi Tech. 40, 848–870. doi: 10.1108/LHT-07-2021-0227

[ref39] Sankaranarayanan R. Nene B. M. Shastri S. S. Jayant K. Muwonge R. Budukh A. M. . (2009). HPV screening for cervical cancer in rural India. N. Engl. J. Med. 360, 1385–1394. doi: 10.1056/NEJMoa0808516, PMID: 19339719

[ref40] Schiffman M. Castle P. E. Jeronimo J. Rodriguez A. C. Wacholder S. (2007). Human papillomavirus and cervical cancer. Lancet 370, 890–907. doi: 10.1016/S0140-6736(07)61416-0, PMID: 17826171

[ref41] Schiffman M. Wentzensen N. (2013). Human papillomavirus infection and the multistage carcinogenesis of cervical cancer. Cancer Epidemiol. Biomarkers Prev. 22, 553–560. doi: 10.1158/1055-9965.EPI-12-1406 23549399 PMC3711590

[ref42] Shannon B. Yi T. Perusini S. Gajer P. Ma B. Humphrys M. . (2017). Association of HPV infection and clearance with cervicovaginal immunology and the vaginal microbiota. Mucosal Immunol. 10, 1310–1319. doi: 10.1038/mi.2016.129, PMID: 28120845 PMC5526752

[ref43] Singh D. Vignat J. Lorenzoni V. Eslahi M. Ginsburg O. Lauby-Secretan B. . (2023). Global estimates of incidence and mortality of cervical cancer in 2020: a baseline analysis of the WHO global cervical Cancer elimination initiative. Lancet Glob. Health 11, e197–e206. doi: 10.1016/S2214-109X(22)00501-0, PMID: 36528031 PMC9848409

[ref44] Stoler M. H. Rhodes C. R. Whitbeck A. Wolinsky S. M. Chow L. T. Broker T. R. (1992). Human papillomavirus type 16 and 18 gene expression in cervical neoplasias. Hum. Pathol. 23, 117–128. doi: 10.1016/0046-8177(92)90232-R, PMID: 1310950

[ref45] Sung H. Ferlay J. Siegel R. L. Laversanne M. Soerjomataram I. Jemal A. . (2021). Global cancer statistics 2020: GLOBOCAN estimates of incidence and mortality worldwide for 36 cancers in 185 countries. CA Cancer J. Clin. 71, 209–249. doi: 10.3322/caac.21660, PMID: 33538338

[ref46] Thomas S. E. Kerry-Smith J. Plummer S. F. Bate J. P. John D. A. Lawrence E. . (2024). The ability of the Lab4 probiotic consortium to impact upon the functionality of serum deprived human keratinocytes in vitro. Front. Microbiolomes 3:1488650. doi: 10.3389/frmbi.2024.1488650 PMC12993548 41853543

[ref47] Valenti P. Rosa L. Capobianco D. Lepanto M. S. Schiavi E. Cutone A. . (2018). Role of lactobacilli and lactoferrin in the mucosal cervicovaginal defense. Front. Immunol. 9:376. doi: 10.3389/fimmu.2018.00376, PMID: 29545798 PMC5837981

[ref48] Wentzensen N. Schiffman M. Palmer T. Arbyn M. (2016). Triage of HPV positive women in cervical cancer screening. J. Clin. Virol. 76, S49–S55. doi: 10.1016/j.jcv.2015.11.015, PMID: 26643050 PMC4789103

[ref49] Zhang X.-L. Zheng Y. Xia M.-L. Wu Y.-N. Liu X.-J. Xie S.-K. . (2020). Knowledge domain and emerging trends in vinegar research: a bibliometric review of the literature from WoSCC. Food Secur. 9:166. doi: 10.3390/foods9020166, PMID: 32050682 PMC7074530

