# Supplementary material for: Vaginal bacteria and cervical cancer: a bibliometric analysis of trends and themes
Source: Front Microbiol. 2025 Jul 30;16:1615944. doi: 10.3389/fmicb.2025.1615944 (PMC12343664; doi:10.3389/fmicb.2025.1615944)
Supplement: Supplementary file 1 [file Table_1.docx]

**Table S1 Publication and Citation Profiles of Leading Countries**

| **Country** | **Articles** | **Freq** | **SCP** | **MCP** | **MCP_Ratio** | **TP** | **TP_rank** | **TC** | **TC_rank** | **Average Citations** |
| --- | --- | --- | --- | --- | --- | --- | --- | --- | --- | --- |
| CHINA | 111 | 0.298 | 102 | 9 | 0.081 | 320 | 2 | 1072 | 2 | 9.7 |
| USA | 70 | 0.188 | 47 | 23 | 0.329 | 331 | 1 | 2328 | 1 | 33.3 |
| UK | 17 | 0.046 | 6 | 11 | 0.647 | 59 | 5 | 701 | 3 | 41.2 |
| BRAZIL | 16 | 0.043 | 12 | 4 | 0.25 | 68 | 3 | 162 | 11 | 10.1 |
| INDIA | 13 | 0.035 | 11 | 2 | 0.154 | 41 | 6 | 71 | 18 | 5.5 |
| MEXICO | 11 | 0.03 | 9 | 2 | 0.182 | 67 | 4 | 316 | 5 | 28.7 |
| TURKEY | 11 | 0.03 | 10 | 1 | 0.091 | 30 | 13 | 150 | 13 | 13.6 |
| SWEDEN | 10 | 0.027 | 5 | 5 | 0.5 | 38 | 7 | 310 | 6 | 31 |
| KOREA | 9 | 0.024 | 8 | 1 | 0.111 | 36 | 11 | 244 | 8 | 27.1 |
| ITALY | 8 | 0.022 | 7 | 1 | 0.125 | 36 | 10 | 200 | 10 | 25 |
| BELGIUM | 7 | 0.019 | 2 | 5 | 0.714 | 24 | 16 | 667 | 4 | 95.3 |
| JAPAN | 7 | 0.019 | 7 | 0 | 0 | 14 | 21 | 156 | 12 | 22.3 |
| NETHERLANDS | 7 | 0.019 | 7 | 0 | 0 | 28 | 14 | 66 | 20 | 9.4 |
| POLAND | 7 | 0.019 | 6 | 1 | 0.143 | 34 | 12 | 89 | 17 | 12.7 |
| CANADA | 6 | 0.016 | 3 | 3 | 0.5 | 37 | 8 | 102 | 16 | 17 |
| GERMANY | 5 | 0.013 | 3 | 2 | 0.4 | 26 | 15 | 36 | 22 | 7.2 |
| SOUTH AFRICA | 4 | 0.011 | 1 | 3 | 0.75 | 37 | 9 | 70 | 19 | 17.5 |
| AUSTRALIA | 3 | 0.008 | 3 | 0 | 0 | 22 | 17 | 209 | 9 | 69.7 |
| IRAN | 3 | 0.008 | 3 | 0 | 0 | 13 | 22 | 126 | 15 | 42 |
| NIGERIA | 3 | 0.008 | 1 | 2 | 0.667 | 20 | 18 | 27 | 26 | 9 |

Articles: Publications of Corresponding Authors only. Freq: Frequence of Total Publications. MCP_Ratio: Proportion of Multiple Country Publications. TP: Total Publications. TP_rank: Rank of Total Publications. TC: Total Citations. TC_rank: Rank of Total Citations. Average Citations: The average number of citations per publication.

**Table S2 Publication and Citation Profiles of High-Impact Authors**

| **Author** | **h_index** | **g-index** | **m-index** | **PY_start** | **TP** | **TP_Frac** | **TP_rank** | **TC** | **TC_rank** |
| --- | --- | --- | --- | --- | --- | --- | --- | --- | --- |
| HERBST-KRALOVETZ MM | 7 | 7 | 1 | 2018 | 7 | 1.26 | 2 | 436 | 2 |
| LANIEWSKI P | 7 | 7 | 1 | 2018 | 7 | 1.26 | 2 | 436 | 2 |
| CHASE DM | 6 | 6 | 0.857 | 2018 | 6 | 0.93 | 4 | 422 | 4 |
| KYRGIOU M | 5 | 5 | 0.294 | 2008 | 5 | 0.86 | 5 | 903 | 1 |
| ZHANG Y | 5 | 8 | 0.833 | 2019 | 8 | 1.01 | 1 | 115 | 10 |
| ROE DJ | 4 | 4 | 0.571 | 2018 | 4 | 0.62 | 7 | 378 | 5 |
| WANG L | 4 | 5 | 0.333 | 2013 | 5 | 0.71 | 5 | 63 | 13 |
| BHATTARAKOSOL P | 3 | 3 | 1 | 2022 | 3 | 0.45 | 12 | 12 | 20 |
| BOON ME | 3 | 4 | 0.158 | 2006 | 4 | 0.78 | 7 | 60 | 14 |
| COLBERT LE | 3 | 3 | 0.6 | 2020 | 3 | 0.21 | 12 | 22 | 19 |
| CUI HY | 3 | 3 | 0.429 | 2018 | 3 | 0.45 | 12 | 238 | 9 |
| DENNY L | 3 | 3 | 0.15 | 2005 | 3 | 0.62 | 12 | 343 | 7 |
| GODOY-VITORINO F | 3 | 4 | 0.429 | 2018 | 4 | 0.64 | 7 | 97 | 11 |
| HEINTZ APM | 3 | 4 | 0.158 | 2006 | 4 | 0.78 | 7 | 60 | 14 |
| HERRERO R | 3 | 3 | 0.115 | 1999 | 3 | 0.32 | 12 | 375 | 6 |
| KOBETZ E | 3 | 3 | 0.2 | 2010 | 3 | 0.58 | 12 | 33 | 17 |
| KUHN L | 3 | 3 | 0.15 | 2005 | 3 | 0.62 | 12 | 343 | 7 |
| LARSSON PG | 3 | 3 | 0.083 | 1989 | 3 | 0.7 | 12 | 54 | 16 |
| LI C | 3 | 4 | 0.429 | 2018 | 4 | 0.64 | 7 | 70 | 12 |
| LI L | 3 | 3 | 0.6 | 2020 | 3 | 0.34 | 12 | 28 | 18 |

H_index: The h-index of the authors, which measures both the productivity and citation impact of the authors. g_index: The g-index of the authors. m_index: The m-index of the authors, which is the h-index divided by the number of years since the first published paper. TP: Total Publications. TP_rank: Rank of Total Publications. TC: Total Citations. TC_rank: Rank of Total Citations. Average Citations: The average number of citations per publication. PY_start: Publication Year Start, indicating the year the journal started publication.

**Table S3 Bibliometric Indicators of High-Impact Journals**

| **Journal** | **H_index** | G_index | **M_index** | **IF 2023** | **JCR 2023** | **TP** | **TP_rank** | **TC** | **TC_rank** | **PY_start** |
| --- | --- | --- | --- | --- | --- | --- | --- | --- | --- | --- |
| BMC INFECTIOUS DISEASES | 8 | 10 | 0.5 | 3.4 | 2 | 10 | 4 | 248 | 5 | 2009 |
| FRONTIERS IN CELLULAR AND INFECTION MICROBIOLOGY | 8 | 12 | 1.333 | 4.6 | 1 | 15 | 1 | 156 | 13 | 2019 |
| PLOS ONE | 8 | 15 | 0.5 | 2.9 | 1 | 15 | 2 | 517 | 1 | 2009 |
| SCIENTIFIC REPORTS | 7 | 12 | 0.7 | 3.8 | 1 | 12 | 3 | 368 | 3 | 2015 |
| INTERNATIONAL JOURNAL OF GYNECOLOGICAL CANCER | 5 | 7 | 0.263 | 4.1 | 1 | 7 | 5 | 105 | 21 | 2006 |
| JOURNAL OF MEDICAL VIROLOGY | 5 | 6 | 0.5 | 6.8 | 1 | 6 | 7 | 96 | 28 | 2015 |
| AMERICAN JOURNAL OF OBSTETRICS AND GYNECOLOGY | 4 | 4 | 0.111 | 8.7 | 1 | 4 | 15 | 252 | 4 | 1989 |
| SEXUALLY TRANSMITTED INFECTIONS | 4 | 5 | 0.182 | 3.6 | 2 | 5 | 14 | 117 | 17 | 2003 |
| ACTA CYTOLOGICA | 3 | 5 | 0.2 | 1.6 | 3 | 5 | 10 | 38 | 79 | 2010 |
| ASIAN PACIFIC JOURNAL OF CANCER PREVENTION* | 3 | 3 | 0.231 | NA | NA | 3 | 21 | 28 | 108 | 2012 |
| DIAGNOSTIC CYTOPATHOLOGY | 3 | 5 | 0.176 | 1 | 4 | 5 | 11 | 59 | 47 | 2008 |
| EUROPEAN JOURNAL OF OBSTETRICS & GYNECOLOGY AND REPRODUCTIVE BIOLOGY | 3 | 3 | 0.188 | 2.1 | 2 | 3 | 25 | 54 | 53 | 2009 |
| FRONTIERS IN MICROBIOLOGY | 3 | 6 | 0.3 | 4 | 2 | 6 | 6 | 99 | 22 | 2015 |
| GYNECOLOGIC ONCOLOGY | 3 | 3 | 0.158 | 4.5 | 1 | 3 | 27 | 182 | 9 | 2006 |
| JOURNAL OF MEDICAL MICROBIOLOGY | 3 | 5 | 0.07 | 2.4 | 3 | 5 | 13 | 39 | 78 | 1982 |
| JOURNAL OF OBSTETRICS AND GYNAECOLOGY | 3 | 5 | 0.2 | 0.9 | 4 | 6 | 8 | 24 | 128 | 2010 |
| JOURNAL OF WOMENS HEALTH | 3 | 3 | 0.176 | 3 | 1 | 3 | 30 | 13 | 202 | 2008 |
| MICROBIAL PATHOGENESIS | 3 | 5 | 0.333 | 3.3 | 2 | 6 | 9 | 28 | 109 | 2016 |
| SEXUALLY TRANSMITTED DISEASES | 3 | 3 | 0.158 | 2.4 | 3 | 3 | 34 | 215 | 8 | 2006 |
| VIROLOGY JOURNAL | 3 | 4 | 0.333 | 4 | 2 | 4 | 19 | 38 | 83 | 2016 |

H_index: The h-index of the journal, which measures both the productivity and citation impact of the publications. IF: Impact Factor, indicating the average number of citations to recent articles published in the journal. JCR_Quartile: The quartile ranking of the journal in the Journal Citation Reports, indicating the journal's ranking relative to others in the same field (Q1: top 25%, Q2: 25%-50%, Q3: 50%-75%, Q4: bottom 25%). TP: Total Publications. TP_rank: Rank of Total Publications. TC: Total Citations. TC_rank: Rank of Total Citations. Average Citations: The average number of citations per publication. PY_start: Publication Year Start, indicating the year the journal started publication. *:Not included in SCI list now

**Table S4** The Most Cited Publications

| **Paper** | **DOI** | **Total Citations** | **TC per Year** | **Normalized TC** |
| --- | --- | --- | --- | --- |
| ARBYN M, 2008, BMJ-BRIT MED J | 10.1136/bmj.a1284 | 511 | 30.06 | 4.61 |
| MITRA A, 2015, SCI REP-UK | 10.1038/srep16865 | 304 | 30.40 | 5.34 |
| DE SANJOSÉ S, 2018, BEST PRACT RES CL OB | 10.1016/j.bpobgyn.2017.08.015 | 280 | 40.00 | 7.04 |
| AUDIRAC-CHALIFOUR A, 2016, PLOS ONE | 10.1371/journal.pone.0153274 | 253 | 28.11 | 7.49 |
| CASTLE PE, 2001, CANCER EPIDEM BIOMAR | NA | 188 | 7.83 | 1.83 |
| MYER L, 2005, J INFECT DIS | 10.1086/462427 | 183 | 9.15 | 1.64 |
| WOOD BR, 1998, BIOSPECTROSCOPY | 3.0.CO;2-R" target="_blank">10.1002/(SICI)1520-6343(1998)4:23.0.CO;2-R | 174 | 6.44 | 1.00 |
| LANIEWSKI P, 2018, SCI REP-UK | 10.1038/s41598-018-25879-7 | 159 | 22.71 | 4.00 |
| USYK M, 2020, PLOS PATHOG | 10.1371/journal.ppat.1008376 | 147 | 29.40 | 7.54 |
| FALK L, 2005, SEX TRANSM INFECT | 10.1136/sti.2004.010439 | 145 | 7.25 | 1.30 |

TC：Total Citations.
